# Case Report: Successful treatment of late-onset immune checkpoint inhibitor-associated membranous nephropathy in a patient with advanced renal cell carcinoma

**DOI:** 10.3389/fimmu.2022.898811

**Published:** 2022-07-28

**Authors:** Praveen Ratanasrimetha, Vikas D. Reddy, Jaya Kala, Amanda Tchakarov, William F. Glass, Pavlos Msaouel, Jamie S. Lin

**Affiliations:** ^1^ Section of Nephrology, Division of Internal Medicine, University of Texas MD Anderson Cancer Center, Houston, TX, United States; ^2^ Division of Renal Diseases and Hypertension, Department of Internal Medicine, University of Texas Health Science Center at Houston, McGovern Medical School, Houston, TX, United States; ^3^ Department of Pathology and Laboratory Medicine, University of Texas Health Science Center McGovern Medical School, Houston, TX, United States; ^4^ Department of Genitourinary Medical Oncology, Division of Cancer Medicine, The University of Texas MD Anderson Cancer Center, Houston, TX, United States; ^5^ Department of Translational Molecular Pathology, Division of Pathology and Laboratory Medicine, The University of Texas MD Anderson Cancer Center, Houston, TX, United States

**Keywords:** immune checkpoint inhibitor (ICI), immune related adverse events (irAEs), membranous nephropathy (MN), proteinuria, rituximab, case report, renal cell carcinoma (RCC)

## Abstract

**Background:**

Diagnosing immune checkpoint inhibitor (ICI)-associated nephritis can be challenging since it is a rare complication of therapy, associated with a spectrum of immune-mediated pathologies, and can present months after ICI therapy discontinuation (i.e., late-onset). ICIs are increasingly administered in combination with other cancer therapies with associated nephrotoxicity, further obfuscating the diagnosis of ICI-associated nephritis. In this report, we describe the first suspected case of late-onset ICI-associated membranous nephropathy (MN) in a patient with metastatic clear cell renal cell carcinoma (RCC) who had discontinued ICI therapy 6 months prior to presentation. Prompt recognition of the suspected late-onset immune-related adverse event (irAE) resulted in the successful treatment of MN and continuation of RCC therapy.

**Case presentation:**

A 57-year-old man with metastatic clear cell RCC was responsive to third-line RCC therapy with lenvatinib (oral TKI) and everolimus (oral mTOR inhibitor) when he presented with nephrotic range proteinuria and acute kidney injury (AKI). His kidney biopsy revealed probable secondary MN with subendothelial and mesangial immune complex deposits and negative staining for both phospholipase A2 receptor (PLA2R) and thrombospondin type-1 domain-containing 7A (THSD7A). While a diagnosis of paraneoplastic MN could not be excluded, the patient was responding to cancer therapy and had tumor regression. However, 6 months prior to presentation, the patient had received pembrolizumab, an ICI, with his first-line RCC treatment. Due to concern that the patient may be presenting with late-onset ICI-associated MN, he was effectively treated with rituximab, which allowed for his continued RCC therapy.

**Conclusion:**

This report highlights the first case of suspected late-onset ICI-associated MN and the increasing complexity of recognizing renal irAEs. With the growing indications for the use of ICIs in combination with other cancer therapies, recognizing the various presentations of ICI-immune nephritis can help guide patient management and treatment.

## Background

Immune checkpoint inhibitors (ICIs) are a novel class of immunotherapy drugs that work by blocking intrinsic negative regulators of the immune system, thus increasing immune activity and enhancing antitumor responses. These drugs are also frequently associated with autoimmune effects in normal tissues defined as immune-related adverse events (irAEs). Renal irAEs are infrequent, occurring in only 2%–5% of those receiving ICI therapy (1, 2). While acute interstitial nephritis (AIN) is the predominant renal lesion associated with ICI therapy, glomerular diseases such as membranous nephropathy (MN) can also develop (2, 3). In patients with renal cell carcinoma (RCC), therapy with ICIs, tyrosine kinase inhibitors (TKIs), and mammalian target of rapamycin (mTOR) inhibitors are used in combination or as monotherapy for RCC treatment. Thus, nephrotoxicity from TKIs, mTOR inhibitors, and ICI therapy can present similarly with proteinuria and acute kidney injury (AKI). Furthermore, irAEs can present several months after ICI therapy has been discontinued (i.e., late onset), obfuscating the diagnosis (4–7). In this report, we describe a suspected case of late-onset ICI-associated MN in a patient with metastatic clear cell RCC who had discontinued ICI therapy 6 months prior to presentation. He was successfully treated with rituximab and able to resume RCC therapy.

## Case presentation

**Figure 1:**
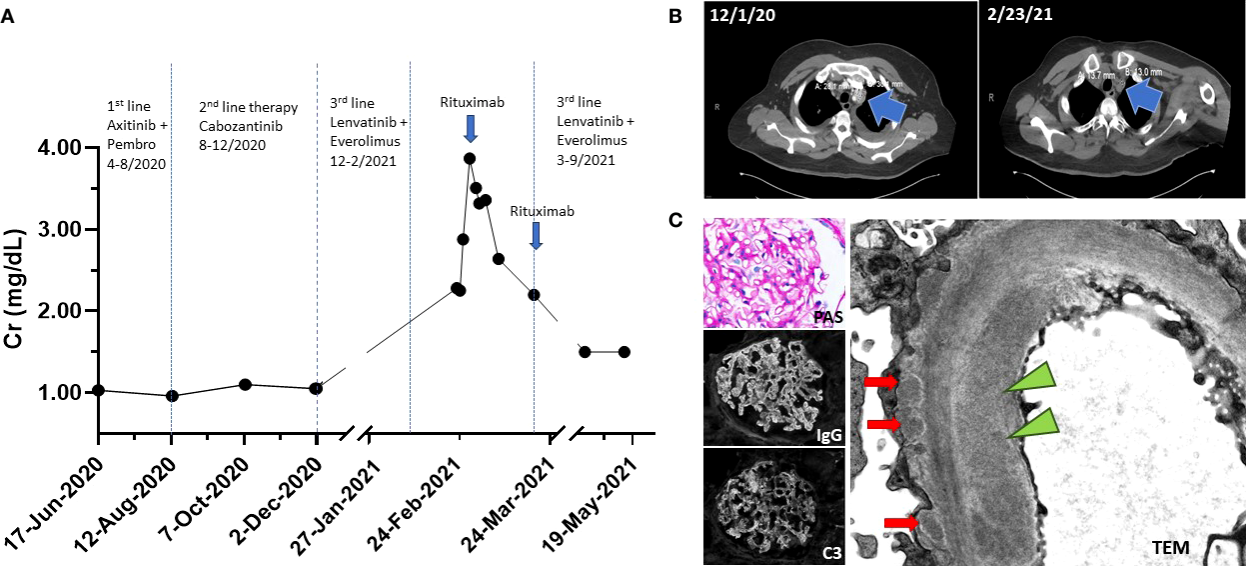
**(A)** Timeline of events. Patient progressed on first- and second-line RCC therapy. Patient was on third-line therapy at the time of ED presentation. Creatinine peaked at 3.83 mg/dl. Rituximab 1 g IV was administered on days 1 and 15 (blue arrow). **(B)** CT imaging on 1 December 2020 showed left superior mediastinal lymph node measuring 28 mm × 30 mm; CT imaging on 23 February 2021 showed a decreased size of left superior mediastinal lymph node measuring 14 mm × 13 mm. **(C)** Renal biopsy. (Top left) Periodic acid-Schiff (PAS) stain shows mildly prominent capillary walls and mesangial expansion. (Middle left) Diffuse granular capillary wall immunoglobulin G (IgG) deposits. (Bottom left) C3 capillary deposits that vary in abundance and intensity. (Right) Electron micrograph images reveal subendothelial electron dense deposit (green triangle) and subepithelial electron dense deposits (red arrows).

A 57-year-old man with metastatic high-grade clear cell RCC presented to the Emergency Department (ED) with complaints of diarrhea and poor oral intake, and was found to have AKI on laboratory analysis. The patient had been diagnosed with localized left-sided high-grade clear cell RCC 5 years prior, for which he underwent nephrectomy. His disease subsequently recurred to the bones, lungs, bilateral adrenal glands, lymph nodes, and brain necessitating stereotactic brain radiosurgery and bilateral pleural catheter placement for malignant pleural effusion drainage. At presentation, he was on third-line combination therapy with lenvatinib (oral TKI) and everolimus (oral mTOR inhibitor). He had previously progressed on a 4-month course of first-line therapy with pembrolizumab (ICI) plus axitinib (oral TKI), which was discontinued 6 months prior to presentation, and a 3-month course of second-line cabozantinib (oral TKI) monotherapy, which was discontinued 3 months prior to presentation (**Figure 1A**). His other past medical history included a diagnosis of hypertension that was controlled with amlodipine 10 mg daily and carvedilol 6.25 mg q 12 h. He also had a history of hypothyroidism that was treated with levothyroxine 150 mcg daily.

His pertinent physical exam findings included decreased bibasilar breath sounds with well-positioned pleural catheters and 2+ bilateral pitting pedal edema. He was started on empiric antibiotics and intravenous (IV) fluids. Laboratory results were notable for a serum creatinine (Cr) of 2.28 mg/dl (201.6 μmol/L) from a baseline serum creatinine of 0.96 mg/dl (84.88 μmol/L), or AKI stage 2 (Cr 2.0–2.9 times baseline) by Kidney Disease Improving Global Outcomes (KDIGO) criteria (8). He had an albumin level of 2.0 g/dl (20 g/L); normal range, 3.5–5.5 g/dl (35-55 g/L). His urinalysis revealed a specific gravity of 1.023, protein ≥ 500 mg/dl, 1 white blood cell/high power field (HPF), 0 red blood cell/HPF, and 1 hyaline cast. A 24-h urine collection revealed 2.85 g of protein. A non-contrast CT scan confirmed colitis and, notably, decreased tumor size of his metastatic disease (**Figure 1B**). Due to his near-nephrotic range proteinuria, serologies were sent and found to be negative for anti-glomerular basement membrane, anti-double-stranded DNA, anti-nuclear antibodies, and anti-neutrophil cytoplasmic antibodies. Complement levels were also normal.

The differential diagnosis causing his AKI was broad due to his complicated medical history and clinical presentation and included acute tubular necrosis (ATN) secondary to colitis, infection, TKI or mTOR therapy, thrombotic microangiopathy (TMA) secondary to TKI therapy, AIN secondary to antibiotics or ICI therapy, and/or GN secondary ICI therapy or cancer. With supportive management, his kidney function continued to deteriorate, and the patient agreed to undergo a kidney biopsy. Light microscopy revealed enlarged glomeruli with subtle basement membrane spikes on silver stain, without mesangial or endocapillary hypercellularity, fibrinoid necrosis, or hyaline thrombi. Mild arterial and arteriolar sclerosis, and mild acute tubular epithelial injury were also present. By immunofluorescence microscopy, there was global granular capillary loop staining for immunoglobulin (Ig) G and C3, as well as kappa and lambda light chain. There was no significant staining for IgA, IgM, or C1q. Electron microscopy (EM) revealed glomerular basement membrane thickening with associated subepithelial electron-dense deposits. Rare segmental mesangial and subendothelial deposits were also present (**Figure 1C**). A diagnosis of secondary MN was favored due to the presence of subendothelial deposits that are only seen in cases of secondary MN. Staining for antibodies to phospholipase A2 receptor (PLA2R) and thrombospondin type-1 domain-containing 7A (THSD7A) were both negative (9). Though the patient had evidence of tumor regression on therapy, a diagnosis of paraneoplastic MN remained a possibility; however, continued RCC therapy for treatment of possible paraneoplastic MN was not an option due to the patients declining renal function. Due to the patient’s history of ICI therapy, we suspected that the patient may be presenting with late-onset ICI-associated MN from his first-line therapy with pembrolizumab.

After discussing the biopsy results with the patient and the possibility that he may be presenting with late-onset ICI-associated MN from his prior RCC therapy, the patient consented to therapy with rituximab (1 g IV, day 1), a monoclonal antibody targeting CD20 on B cells. He tolerated the therapy and had no adverse issues associated with rituximab. His serum Cr stabilized and improved to Cr 2.64 mg/dl (233.43 μmol/L) at discharge from a peak Cr 3.83 mg/dl (338.57 μmol/L). Two weeks after discharge, his follow-up laboratory results showed a Cr 1.50 mg/dl (132.63 μmol/L) with a serum albumin of 2.7 g/dl (27 g/L) and a urine protein-to-creatinine ratio of 3.15 g/g. He received his second dose of rituximab (1 g IV, day 14) and was concurrently restarted on lenvatinib and everolimus. Five months after his ED presentation, he ultimately succumbed to complications from his metastatic RCC.

## Discussion and conclusions

Cancer immunotherapies and targeted therapies are associated with a spectrum of adverse kidney side effects. With the widespread use and combination of cancer therapies, the incidence of adverse renal events may become more common and difficult to diagnose (10, 11). We present a patient with advanced RCC found to have suspected late-onset ICI-associated MN that responded to rituximab therapy. In this discussion, we will review the kidney side effects associated with RCC therapies and indications and considerations for ICI-associated MN treatment.

Kidney side effects from cancer immunotherapies and targeted therapies will frequently present with similar clinical findings (i.e., proteinuria and AKI) requiring a biopsy for diagnosis. The patient presented on third-line RCC therapy with lenvatinib (oral TKI) and everolimus (oral mTOR inhibitor), which, in itself, has known kidney toxicities (12, 13). Lenvatinib is a multi-TKI that notably targets vascular endothelial growth factor (VEGF) receptor 1, 2, and 3 among other tyrosine kinase receptors. Blockade of VEGF receptors on glomerular podocytes decreases VEGF secretion to glomerular endothelial cells resulting in loss of endothelial fenestration and microthrombi development or TMA (14). Everolimus is an mTOR inhibitor. MTOR signaling is not only involved with the VEGF pathway, but mTOR signaling in glomerular podocytes regulates the migration and adhesion of podocytes to the glomerular basement membrane. Dysregulation of mTOR homeostasis results in podocyte cytoskeletal remodeling, foot process effacement, and, consequently, breakdown of the glomerular filtration barrier resulting in proteinuria (15, 16). Dysregulation of mTOR has also been associated with both beneficial and pathogenic effects on glomerular health including the induction of focal segmental glomerulosclerosis (FSGS) or reduction of proteinuria and mesangial and endocapillary proliferation in patients with IgA nephropathy (17, 18). TKIs and mTOR inhibitors have distinct effects on podocyte biology that can present as proteinuria. Without the kidney biopsy, toxicity from lenvatinib and everolimus therapy in our patient could not have been excluded from the differential and would have impacted the decision to resume his cancer therapy.

Usage of pembrolizumab (ICI) plus axitinib (oral TKI) for advanced RCC was introduced in April 2019 (19, 20). Immune checkpoint proteins such as cytotoxic T lymphocyte antigen-4 (CTLA-4) and programmed cell death protein-1 (PD-1) are negative costimulatory molecules that attenuate T-cell activation. ICIs like pembrolizumab target and block PD-1 removing the inhibitory signals of T-cell activation, enabling tumor-reactive T cells to mount an antitumor response. Increasing T-cell activity, however, also increases the production of inflammatory cytokines and anti-humoral response, which can result in the development of autoimmune complications characterized as irAEs (21). Renal irAEs occur infrequently and represent a spectrum of immune-mediated diseases. The most common pathologic lesion in ICI-related AKI is AIN. However, *de novo* vasculitis and glomerular lesions have also been reported, including pauci-immune glomerulonephritis, IgA nephropathy, C3 glomerulopathy, and MN (2, 3). ICI therapy can also trigger reactivation of autoimmune diseases such as primary PLA2R-positive MN (22).

Our patient had biopsy-proven MN. While MN can be primary or secondary, staining for anti-PLA2R and THSD7A, which account for 85% of patients who develop primary MN, was negative and the presence of subendothelial and mesangial deposits on EM strongly suggested that he did not have primary MN (9). *De novo* PLA2R positive MN has been reported in patients with cancer, where these patients exhibit persistent proteinuria after tumor resection (23, 24).

MN can also present as a paraneoplastic symptom. Paraneoplastic MN is common in malignancies of the lung, prostate, and gastrointestinal tract, but its association with clear cell RCC is rare (25, 26). Paraneoplastic diseases are generally reported within a year of cancer diagnosis but development years after cancer diagnosis have also been reported (27). Despite evidence of tumor regression in our patient and the possibility that he may be presenting with paraneoplastic MN, our patient had an additional risk factor that could have also led to the development of MN and that was his prior RCC therapy with pembrolizumab.

ICI-associated MN is rarely reported as a renal irAE, with only 7 reported cases of MN associated with ICI therapy to date (3, 22, 28–31). Five of these cases were *de novo* MN while 2 of these cases were associated with ICI-induced MN reactivation (**Table 1**). In all 7 cases, the patients were diagnosed with MN while on ICI therapy. However, delayed or late-onset presentations of irAEs are increasingly recognized and hypothesized to be associated with the expansion of CD8^+^ memory T cells from ICI therapy (7). Late-onset irAEs have been observed months to over a year after ICI therapy discontinuation in non-kidney organs (4–6). As indications for ICI therapy expands, this case highlights the importance of considering late-onset irAEs in the differential diagnosis of patients who have previously received ICI therapy (1, 32).

**Table 1:** Clinical characteristics of MN cases associated with ICI therapy.

Case	Age Sex	Primary cancer	ICI therapy	Time from ICI initiation to MN diagnosis	*De Novo* or Reactivation	PLA2R and/or THSD7A status	MN treatment	Renal Outcome	Reference
**1**	60 F	Renal cell carcinoma	Nivolumab (anti-PD-1)	4 months	De novo	PLA2R negative	Glucocorticoid	Complete renal Recovery	Ref (3)
**2**	55 M	Non-small cell lung cancer	Durvalumab (anti-PD-L1)	9 months	Reactivation	PLA2R positive	ACE inhibitor	Partial renal recovery	Ref (28)
**3**	53 F	Ovarian Cancer	Unknown	~9 weeks	De novo	Not reported	Glucocorticoid	Complete renal recovery	Ref (29)
**4**	39 M	Colon Cancer	Unknown	~8.5 weeks	De novo	Not reported	Glucocorticoid	Complete renal recovery	Ref (29)
**5**	60 M	Malignant pleural mesothelioma	Nivolumab (anti-PD-1)	13 months	Reactivation	PLA2R positive	Rituximab	Partial renal recovery	Ref (22)
**6**	74 M	Lung adenocarcinoma	Tislelizumab (anti-PD-1)	~8 months	De novo	PLA2R negative THSD7A positive	Glucocorticoid and Rituximab	Complete renal recovery	Ref (30)
**7**	69 M	Lung adenocarcinoma	Nivolumab (anti-PD-1)	5 months	De novo	PLA2R negative	Glucocorticoid	Partial renal recovery	Ref (31)
**8**	57 M	Renal cell carcinoma	Pembrolizumab (anti-PD-1)	10 months	De novo	PLA2R negative THSD7A negative	Rituximab	Partial renal recovery	Current case

Membranous nephropathy (MN); immune checkpoint inhibitor (ICI); male (M); female (F); angiotensin-converting enzyme (ACE); phospholipase A2 receptor (PLA2R); thrombospondin type- 1 domain-containing 7A (THSD7A).

Given our patient’s laboratory findings and history of ICI therapy, our patient was considered high risk for fulminant renal failure (33). A cytotoxic agent such as cyclophosphamide with corticosteroids is typically recommended for these cases to suppress both T- and B-cell activity. However, due to the patient’s clinical response to cancer therapy, suppression of both T and B cells could negatively impact the immune system’s ability to fight tumor cells. Rituximab, a chimeric monoclonal antibody targeting CD20, has been increasingly shown to be effective in treating antibody-mediated diseases associated with ICI therapy and MN (22, 34, 35). Treatment with rituximab improved his kidney function, and the patient was able to resume third-line RCC treatment with lenvatinib plus everolimus.

Renal irAEs from ICI therapy includes a spectrum of immune-mediated kidney diseases that can present months after ICI therapy completion, obfuscating the diagnosis. This report highlights a suspected case of late-onset ICI-associated MN. With the growing indications for the use of ICIs in combination with other cancer therapies, recognizing the various presentations of ICI-immune nephritis can help guide patient management and treatment.

## Data availability statement

The original contributions presented in the study are included in the article/supplementary material. Further inquiries can be directed to the corresponding author.

## Author contributions

Data curation: VR, PR, JK, AT, and WG. Visualization: VR, PR, and JL. Writing—original draft: PR, VR, PM, and JL. Writing—review and editing: PR, VR, JK, AT, WG, PM, and JL. Project administration, supervision, and conceptualization: JL. All authors have read and approved the manuscript. All authors contributed to the article and approved the submitted version.

## Funding

JL salary support and research reagents are supported by the National Institutes of Health/National Institute of Diabetes and Digestive Kidney Diseases (K08 DK119466), the University of Texas MD Anderson Cancer Center Division of Internal Medicine Immuno-Oncology Toxicity Award Program, and the University of Texas MD Anderson Cancer Center Institutional Research Grant. PM salary support and research reagents are supported by a Career Development Award by the American Society of Clinical Oncology, a Research Award by KCCure, the MD Anderson Khalifa Scholar Award, the Andrew Sabin Family Foundation Fellowship, a Translational Research Partnership Award (KC200096P1) by the United States Department of Defense, an Advanced Discovery Award by the Kidney Cancer Association, the MD Anderson Physician-Scientist Award, and philanthropic donations by Mike and Mary Allen. The University of Texas MD Anderson Cancer Center is supported in part by the National Institutes of Health through Cancer Center Support Grant (P30 CA016672).

## Conflict of interest

PM has received honoraria for service on a Scientific Advisory Board for Mirati Therapeutics, Bristol Myers Squibb, and Exelixis; consulting for Axiom Healthcare Strategies; non-branded educational programs supported by Exelixis and Pfizer; and research funding for clinical trials from Takeda, Bristol Myers Squibb, Mirati Therapeutics, Gateway for Cancer Research, and UT MD Anderson Cancer Center. JK is on the speaker panel for BTG International.

The authors declare that the research was conducted in the absence of any commercial or financial relationships that could be construed as a potential conflict of interest.

## Publisher’s note

All claims expressed in this article are solely those of the authors and do not necessarily represent those of their affiliated organizations, or those of the publisher, the editors and the reviewers. Any product that may be evaluated in this article, or claim that may be made by its manufacturer, is not guaranteed or endorsed by the publisher.

## Abbreviations

AIN, acute interstitial nephritis; AKI, acute kidney injury; CTLA-4, cytotoxic T lymphocyte; FSGS, focal segmental glomerulosclerosis; ICI, immune checkpoint inhibitor; PD-1, programmed cell death protein-1; irAEs, immune-related adverse events; MN, membranous nephropathy; mTOR, mammalian target of rapamycin; PLA2R, phospholipase A2 receptor; RCC, renal cell carcinoma; THSD7A, thrombospondin type-1 domain-containing 7A; TKIs, tyrosine kinase inhibitors; TMA, thrombotic microangiopathy; VEGF, vascular endothelial growth factor.
